# Comparison of anti-Müllerian hormone and antral follicle count in the prediction of ovarian response: a systematic review and meta-analysis

**DOI:** 10.1186/s13048-023-01202-5

**Published:** 2023-06-27

**Authors:** Yang Liu, Zhengmei Pan, Yanzhi Wu, Jiamei Song, Jingsi Chen

**Affiliations:** Department of Reproduction, The Second Affiliated Hospital of Kunming Medical University, Kunming, 650101 China

**Keywords:** Anti-Müllerian hormone, Antral follicle count, Ovarian response, In vitro fertilization, Meta-analysis

## Abstract

**Background:**

Increasingly studies reported that the Anti-Müllerian hormone (AMH) seems to be a promising and reliable marker of functional ovarian follicle reserve, even better than the AFC test. Our study aimed to conduct a meta-analysis to assess the predictive value of AMH and AFC for predicting poor or high response in IVF treatment. An electronic search was conducted, and the following databases were used: PubMed, EMBASE, and the Cochrane Library (up to 7 May 2022). The bivariate regression model was used to calculate the pooled sensitivity, specificity, and area under the receiver operator characteristic (ROC) curve. Subgroup analyses and meta-regression also were used in the presented study. Overall performance was assessed by estimating pooled ROC curves between AMH and AFC.

**Results:**

Forty-two studies were eligible for this meta-analysis. Comparison of the summary estimates for the prediction of poor or high response showed significant difference in performance for AMH compared with AFC [poor (sensitivity: 0.80 vs 0.74, *P* < 0.050; specificity: 0.81 vs 0.85, *P* < 0.001); high (sensitivity: 0.81 vs 0.87, *P* < 0.001)]. However, there were no significant differences between the ROC curves of AMH and AFC for predicting high (*P* = 0.835) or poor response (*P* = 0.567). The cut-off value was a significant source of heterogeneity in the present study.

**Conclusions:**

The present meta-analysis demonstrated that both AMH and AFC have a good predictive ability to the prediction of poor or high responses in IVF treatment.

**Supplementary Information:**

The online version contains supplementary material available at 10.1186/s13048-023-01202-5.

## Background

Controlled ovarian stimulation (COS) is the key to successful assisted reproductive technology (ART). Individualization of COS in in vitro fertilization (IVF) treatments should be based on assessing ovarian reserve and predicting ovarian response for every patient [1]. The starting point is to identify if a patient is likely to have a normal, poor, or high response, and choose the best treatment protocol tailored to this prediction [1]. Patients’ characteristics and biomarkers could accurately predict ovarian response [2]. However, although numerous biochemical measures have been developed to predict IVF outcomes, some biochemical measures, such as estradiol (E^2^), luteinizing hormone (LH), basal follicle-stimulation hormone (FSH), and inhibin concentrations, fluctuate greatly on the day of the menstrual cycle and do not significantly change with decreasing of ovarian reserve, thus they have limited use owing to a low predictive value [3, 4]. Studies have shown that antral follicle count (AFC) is a better indicator to predict ovarian response than other endocrine markers [5, 6].

AMH, a dimeric glycoprotein, is a member of the extended transforming growth factor-β (TGF-β) family [7, 8]. AMH production diminishes as the follicles become FSH-dependent [8, 9]. Serum levels are not affected during the menstrual cycle, are most probably not manipulated by exogenous steroid administration, and are closely correlated with reproductive age [10]. Therefore, AMH has been used to predict poor and high response in IVF. Several studies argued that the level of AMH is a better predictor of ovarian response than the AFC [11]. However, the data remains conflicting and inconsistent [10]. Furthermore, some studies continue to advocate both AFC and AMH as possible predictors of ovarian response [12]. Although Broer and his colleagues [13, 14] have performed meta-analyses in 2009 and 2011 and demonstrated that AMH has at least the same level of accuracy and clinical value for the prediction of poor or excessive response as AFC, the number of the included studies in their meta-analysis were small (*N* = 5–12). Therefore, our study aimed to conduct a meta-analysis that included more eligible studies, to assess the diagnostic value of AMH and AFC for predicting poor or high response in IVF treatment.

## Methods

The present meta-analysis was performed according to the Preferred Reporting Items for Systematic Reviews and Meta-analyses (PRISMA) statement [15].

### Search strategy and data sources

The data sources include these electronic databases: PubMed, EMBASE, and the Cochrane Library (up to 1 May 2022). The following keywords were used: in vitro fertilization (IVF), in vitro fertilization, fertilization in vitro, assisted, or intracytoplasmic in combination with Anti-Mullerian Hormone (AMH), Mullerian-Inhibiting Factor, Mullerian-Inhibitory Substance, Mullerian Inhibiting Hormone, or Antral Follicle Count (AFC). There was no language limitation, and we also retrieved articles by manual screening. A complete search strategy for literature search has provided in Supplementary material.

### Inclusion and exclusion criteria

The inclusion criteria were based on the Population, Intervention, Comparator, Outcomes, and Study designs (PICOS) structure: P): adult infertile women; I) patients receiving COS for IVF/ICSI; C) AMH or AFC to predict ovarian reserve; O) ovarian response including poor or high response; S) prospective design. Besides, if 2 × 2 tables were constructed from the data presented in the paper, the study was included for final analysis in this meta-analysis. Reviews, conference abstracts, case reports, letters, and animal trials were excluded from this study.

### Data extraction

Information was extracted from eligible studies by two authors independently. The following information was included: the authors of the articles, publication year, study location, definition of poor or high response, sample size, true positives (TP), false positives (FP), false negatives (FN), true negatives (TN), and cut-off value. Disagreements were resolved by discussion among all authors.

### Study quality assessment

Our study adopted the Quality Assessment of Diagnostic Accuracy Studies 2 (QUADAS-2) [16] to assess the quality of the included articles, which was the most recommended quality assessment tool for diagnostic accuracy tests. It consists of four main components: patient selection, index test, reference standard, and flow and timing. All components will be assessed for risk of bias, and the first 3 components will also be assessed for clinical applicability. The risk of bias is judged by signature questions, but there are no signature questions for clinical applicability. The “yes”, “no” or “uncertain” answers to the signature questions included in each component may correspond to a bias risk rating of “low”, “high” or “uncertain”. If the answer to all the signature questions in a range is “yes”, then the risk of bias can be assessed as low; If the answer to one of the questions is “no”, the risk of bias is judged to be “high”. The “uncertain” refers to the fact that the literature does not provide detailed information that makes it difficult for the evaluator to make a judgment, and can only be used when the reported data is insufficient.

### Statistical analysis

This meta-analysis used Stata V.14.0 (Stata Corp LP) to conduct all statistical analyses. The Cochrane Q and *I*^2^ statistics were used to test the heterogeneity among all studies. *I*^2^ > 50% indicates the existence of heterogeneity. The bivariate regression model was used to calculate the pooled sensitivity, specificity, and area under the receiver operator characteristic (ROC) curve, and their 95% confidence intervals (CIs). Overall performance was assessed by estimating a pooled ROC curve between AMH and AFC. Furthermore, meta-regression was used to explore the causes of heterogeneity between the studies. Subgroup analyses were performed based on the cut-off value and sample size. Deeks’ funnel plot was used to test publication bias. A two-tailed probability value below 0.05 was regarded as statistically significant.

## Results

### Study selection and study characteristics

**Fig. 1:**
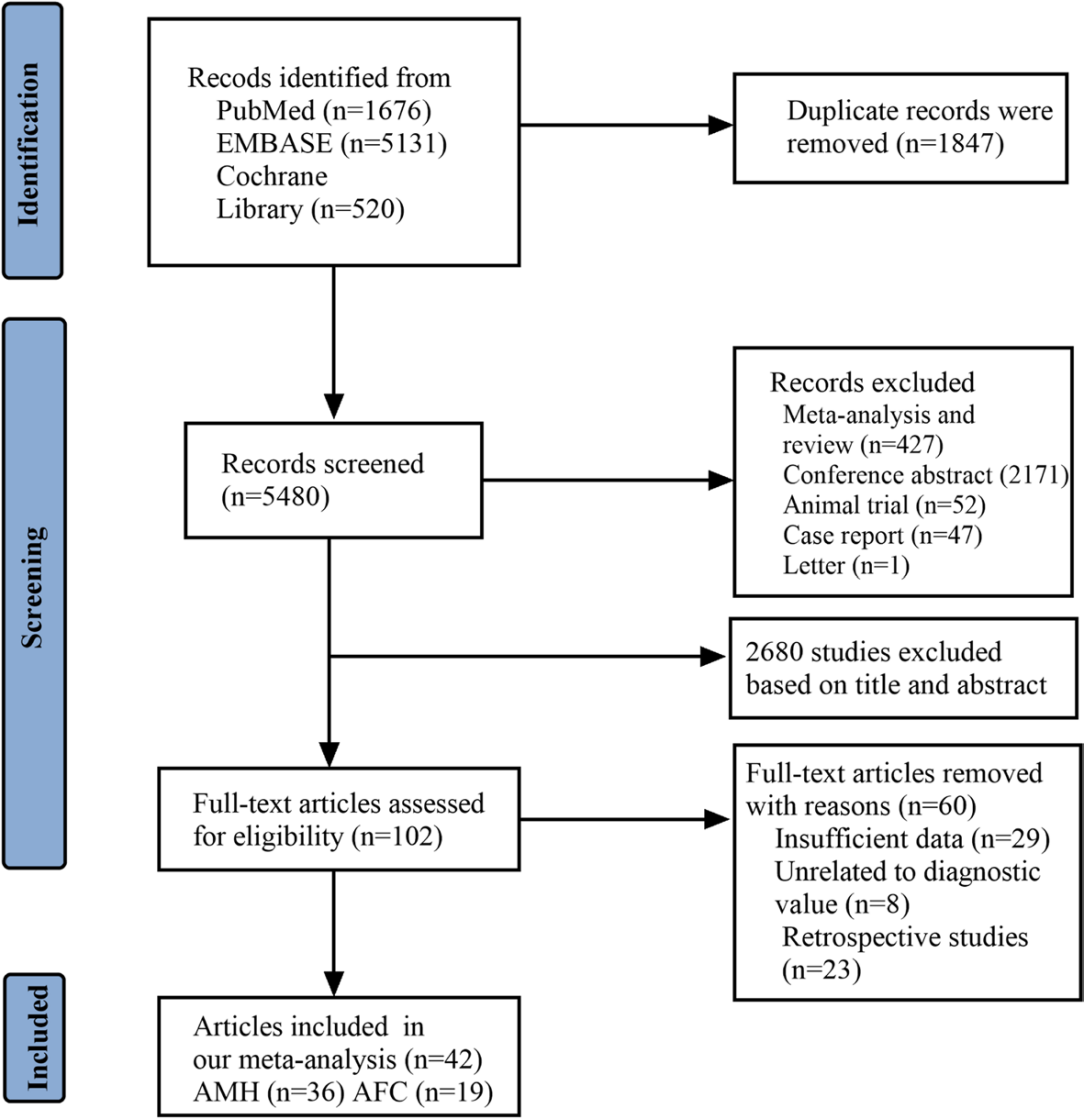
Flow diagram of the process of studies selection

In sum, 7327 articles were identified in electronic and manual searches. However, 1847 articles were excluded for duplication, and another 2698 articles were excluded due to study types (reviews, meeting abstracts, letters, animal trials, and case reports). In addition, 2680 records were excluded after reviewing the title and abstract, and we excluded 60 records after reviewing the full text of 102 articles. Finally, 42 articles [10, 11, 17-56] were included in this meta-analysis (Fig. 1).

The characteristics of the eligible studies are listed in Tables 1 and 2. The sample sizes of participants in each study ranged from 44 to 571, and this meta-analysis included 7190 individuals. Of the 42 studies, all studies were prospective design. The publication year of 42 studies ranged from 2002 to 2021. The included studies were from different countries, including China (*n* = 3), Spain (*n* = 4), the UK (*n* = 7), the USA (*n* = 4), and so on. AMH was used in 29 studies, and AFC in 15 studies in terms of poor response. As for the high response, AMH was used in 13 studies, and AFC in 6 studies.

**Table 1:** Characteristics of the studies included of AMH for predicting ovarian response

Author	Region	Definition of ovarian response	TP	FP	FN	TN	Cut-off
Poor response							
Li et al. 2016 [41]	China	≤ 5 oocytes	23	47	21	321	1.1 ng/ml
Fouda et al. 2010 [27]	Egypt	< 3 follicles	8	18	2	32	0.9 ng/ml
Singh et al. 2013 [51]	India	< 4 oocytes	8	9	2	36	NS
Martínez et al. 2013 [42]	Spain	< 6 oocytes	19	29	8	47	2.31 ng/ml
Baker et al. 2018 [22]	USA	≤ 4 oocytes	20	13	7	120	0.93 ng/ml
Kamel et al. 2014 [57]	Egypt	NS	30	1	5	74	2.8ug/l
Fabregues et al. 2018 [25]	Spain	≤ 3 oocytes	43	77	8	310	NS
Heidar et al. 2015 [30]	Iran	≤ 3 oocytes	16	32	6	134	1.2 ng/ml
Ashrafi et al. 2017 [20]	Iran	≤ 4 oocytes	90	116	32	312	1.05 ng/ml
Neves et al. 2020 [48]	Belgium^a^	≤ 3 oocytes	46	56	4	113	1.00 ng/ml
Islam et al. 2016 [31]	Egypt	≤ 3 oocytes	9	40	6	45	1.4 ng/ml
Baker et al. 2021 [21]	USA, Canada	≤ 4 oocytes	47	43	27	355	0.93 ng/ml
Palhares et al. 2018 [17]	Brazil	≤ 3 oocytes	36	41	9	55	1.5 ng/ml
Jayaprakasan et al. 2010 [34]	UK	≤ 3 oocytes	15	32	0	88	0.99 ng/ml
Tolikas et al. 2011 [18]	Greece	< 4 oocytes	20	18	9	43	2.74 ng/ml
Tremellen et al. 2005 [54]	Australia	≤ 4 oocytes	16	8	4	47	8.1 pmol/l
Kunt et al. 2011 [37]	Turkey	< 5 oocytes	46	14	0	120	2.97 ng/ml
Marca et al. 2007 [38]	Italy	< 4 oocytes	10	3	2	33	0.75 ng/ml
Mutlu et al. 2013 [10]	Turkey	< 4 oocytes	34	20	15	123	0.94 ng/ml
Peñarrubia et al. 2005 [49]	Spain	< 3 follicles	11	2	9	58	4.9 pmol/l
Nardo et al. 2009 [46]	UK	< 4 follicles	13	50	2	101	1.00 ng/ml
Fiçicioglu et al. 2006 [26]	Turkey	< 5 follicles	10	3	1	30	0.25 pg/ml
McIlveen et al. 2007 [43]	UK	≤ 4 oocytes	11	26	2	45	1.25 ng/ml
Muttukrishna et al. 2004 [44]	UK	< 4 follicles	15	14	2	38	0.1 ng/ml
Nakhuda et al. 2007 [45]	USA	NS	20	8	2	36	0.35 ng/ml
Gnoth et al. 2008 [29]	Germany	≤ 4 oocytes	32	58	1	41	1.26 ng/ml
Nelson et al. 2007 [47]	UK	≤ 2 oocytes	14	29	5	292	5 pmol/l
van Rooij et al. 2002 [55]	Netherlands	< 4 oocytes	21	9	14	75	0.3 ng/ml
Lee et al. 2011 [39]	Taiwan	NS	11	16	6	93	0.68 ng/ml
High response							
Li et al. 2016 [41]	China	> 15 oocytes	165	149	38	219	2.6 ng/ml
Akbari Sene et al. 2021 [50]	Iran	> 15 oocytes	31	16	10	43	4.95 ng/ml
Izhar et al. 2021 [32]	Pakistan	NS	50	14	4	208	6.43 ng/ml
Tan et al. 2021 [53]	China	> 15 oocytes	137	15	30	154	3.6 ng/ml
Heidar et al. 2015 [30]	Iran	> 12 oocytes	30	34	23	93	3.40 ng/ml
Ashrafi et al. 2017 [20]	Iran	≥ 15 oocytes	79	129	40	302	2.5 ng/ml
Vembu et al. 2017 [11]	India	≥ 20 oocytes	11	15	2	132	4.85 ng/ml
Neves et al. 2020 [48]	Belgium^a^	> 15 oocytes	13	16	11	179	2.25 ng/ml
Nardo et al. 2009 [46]	UK	> 20 oocytes	14	45	2	104	3.5 ng/ml
Eldar-Geva et al. 2005 [24]	Israel	> 20 oocytes	12	4	5	35	3.5 ng/ml
Aflatoonian et al. 2009 [19]	Iran	> 15oocytes	42	22	3	76	34.5 pmol/l
Lee et al. 2008 [40]	China	NS	19	45	2	196	3.36 ng/ml
Nelson et al. 2007 [47]	UK	≥ 21 oocytes	15	16	10	299	25 pmol/l

*NS* Not stated, *TP* True positive, *FP* False positive, *FN* False negative, *TN* True negative

^a^Region included Belgium, Spain, Germany, Italy

**Table 2:** Characteristics of the studies included of AFC for predicting ovarian response

Author	Region	Definition of ovarian response	TP	FP	FN	TN	Cut-off
Poor response							
Fabregues et al. 2018 [25]	Spain	≤ 3 oocytes	40	61	10	326	NS
Ashrafi et al. 2017 [20]	Iran	≤ 4 oocytes	100	116	22	312	8
Neves et al. 2020 [48]	Belgium^a^	≤ 3 oocytes	42	32	8	137	6
Islam et al. 2016 [31]	Egypt	≤ 3 oocytes	13	34	2	51	7
Palhares et al. 2018 [17]	Brazil	≤ 3 oocytes	36	40	9	56	8
Frattarelli et al. 2003 [28]	USA	< 3 oocytes	7	10	16	234	4
Jayaprakasan et al. 2010 [34]	UK	≤ 3 oocytes	14	14	1	106	10
Tolikas et al. 2011 [18]	Greece	< 4 oocytes	21	12	8	49	5
Mutlu et al. 2013 [10]	Turkey	< 4 oocytes	45	13	4	130	6
Jayaprakasan et al. 2007 [35]	UK	< 4 follicles	5	4	0	91	6
McIlveen et al. 2007 [43]	UK	≤ 4 oocytes	6	14	7	57	5
Bancsi et al. 2004 [23]	Netherlands	< 4 oocytes	22	10	14	74	4
Yong et al. 2003 [56]	UK	< 3 oocytes	1	1	7	37	4
Järvelä et al. 2003 [32]	Canada	< 5 follicles	10	5	2	28	4
Soldevila et al. 2007 [52]	Spain	≤ 5 follicles	75	52	46	154	8
High response							
Izhar et al. 2021 [32]	Pakistan	NS	51	6	3	216	18
Tan et al. 2021 [53]	China	> 15 oocytes	145	19	22	150	18
Ashrafi et al. 2017 [20]	Iran	≥ 15 oocytes	87	116	32	315	15
Neves et al. 2020 [48]	Belgium^a^	≤ 3 oocytes	19	46	5	149	10
Eldar-Geva et al. 2005 [24]	Israel	> 20 oocytes	16	26	1	13	14
Aflatoonian et al. 2009 [19]	Iran	> 15oocytes	40	8	5	90	16

*NS* Not stated, *TP* True positive, *FP* False positive, *FN* False negative, *TN* True negative

^a^Region included Belgium, Spain, Germany, Italy

### Study quality

We adopted the QUADAS-2 to assess the quality of concerning studies (Supplementary material). Regarding risk of bias, 5 studies included consecutive patients, and 37 studies were low risk in index test. Besides, as for applicability of concern, all studies were low risk in both patient selection and index test.

### Accuracy of AMH and AFC for predicting poor response

**Fig. 2:**
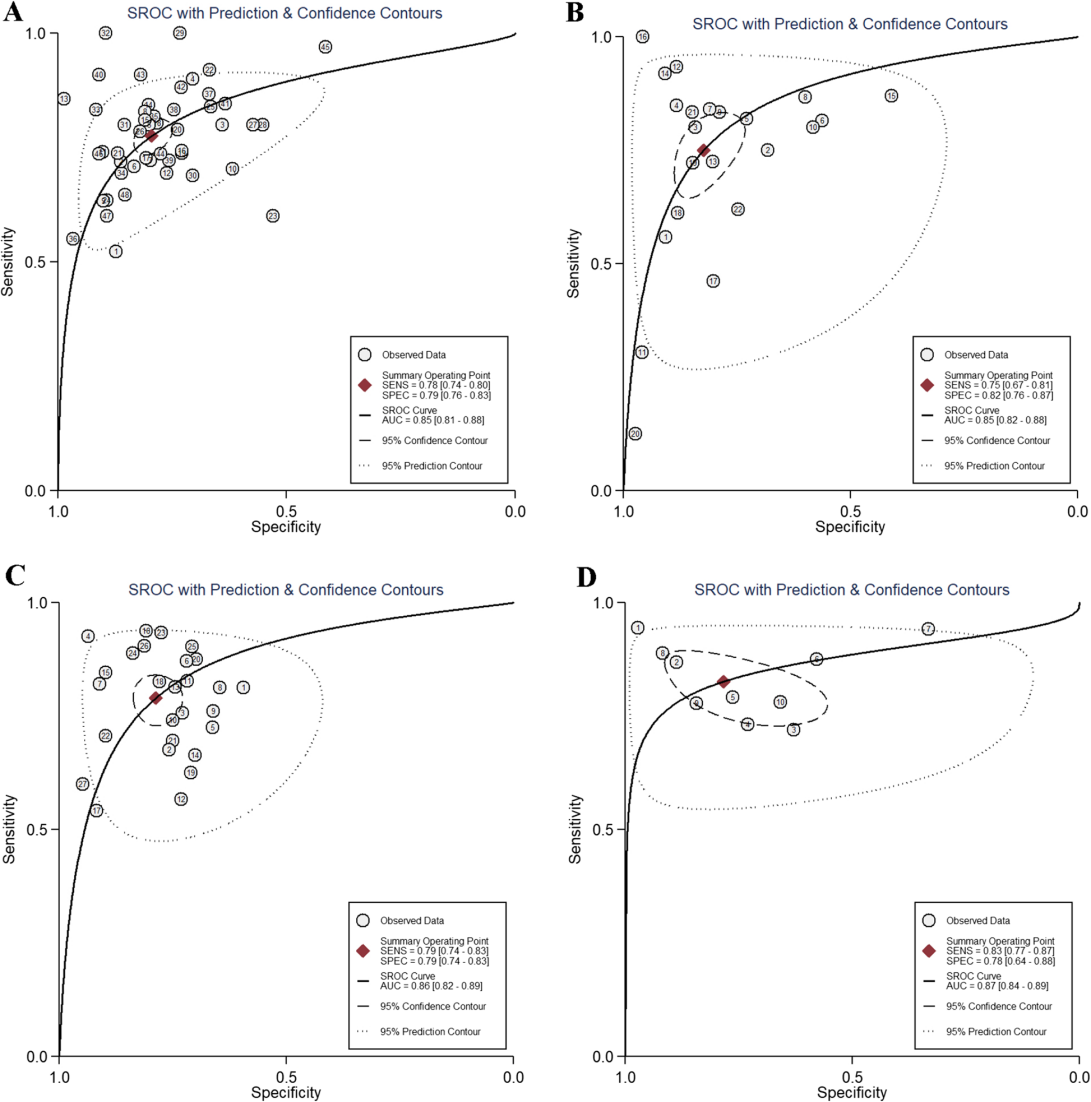
The summary receiver operating characteristic (SROC) curve of AMH and AFC for the prediction of ovarian response. **A** AMH-poor response; **B** AFC- poor response; **C** AMH-high response; **D** AFC-high response

The pooled predictive ability of AMH and AFC for poor response in IVF/ICSI treatments was presented in Table 3. The overall pooled sensitivity and specificity of AMH were 0.80 (95%CI: 0.74–0.85) and 0.81 (95%CI: 0.75–0.85), respectively. The test for heterogeneity demonstrated that there was significant heterogeneity in both sensitivity and specificity (*I*^2^ = 68.26% and 92.43%, respectively). The overall ROC curve was presented in Fig. 2A, and AUC was 0.87 (95%CI: 0.84–0.90). The meta-analysis’s overall pooled sensitivity and specificity of AFC were 0.73 (95%CI: 0.62–0.83) and 0.85 (95%CI: 0.78–0.90), respectively. Heterogeneity was found in both sensitivity and specificity (*I*^2^ = 85.28% and 91.76%, respectively). The overall ROC curve was presented in Fig. 2B, and AUC was 0.87 (95%CI: 0.84–0.90).

**Table 3:** Results of the subgroup analysis

Subgroup	Number (n)	Sensitivity (95%CI)	Specificity (95%CI)	PLR (95%CI)	NLR (95%CI)	DOR (95%CI)
**Cut-off value**						
AMH-poor response						
Overall	29	0.80 (0.74, 0.85)	0.81 (0.75, 0.85)	4.10 (3.20, 5.30)	0.25 (0.19, 0.32)	14.39 (10.26, 20.17)
< 1.00 ng/ml 1	10	0.79 (0.69, 0.89)	0.84 (0.78, 0.89)	4.54 (3.55, 5.83)	0.35 (0.27, 0.44)	16.07 (11.53, 22.39)
≥ 1.00 ng/ml 0	12	0.82 (0.74, 0.90)	0.70 (0.62, 0.77)	2.59 (2.05, 3.28)	0.34 (0.24, 0.49)	8.13 (5.05, 13.09)
AMH-high response						
Overall	13	0.81 (0.75, 0.86)	0.84 (0.77, 0.89)	5.00 (3.40, 7.30)	0.22 (0.16, 0.30)	22.67 (12.85, 40.00)
< 4.00 ng/ml	8	0.75 (0.66, 0.83)	0.80 (0.72, 0.88)	3.63 (2.53, 5.19)	0.34 (0.24, 0.49)	11.83 (5.89, 23.73)
≥ 4.00 ng/ml	3	0.86 (0.76, 0.96)	0.88 (0.79, 0.97)	6.93 (2.56, 18.76)	0.17 (0.06, 0.52)	41.01 (5.36, 313.99)
AFC-poor response						
Overall	15	0.73 (0.62, 0.83)	0.85 (0.78, 0.90)	4.26 (3.23, 5.62)	0.33 (0.22, 0.49)	13.93 (8.53, 22.74)
< 6	7	0.61 (0.44, 0.79)	0.90 (0.84, 0.95)	5.18 (3.41, 7.85)	0.42 (0.24, 0.76)	14.06 (5.93, 33.34)
≥ 6	7	0.83 (0.72, 0.94)	0.79 (0.69, 0.88)	3.60 (2.53, 5.13)	0.27 (0.17, 0.44)	12.60 (6.31, 25.14)
AFC-high response						
Overall	6	0.85 (0.77, 0.91)	0.83 (0.64, 0.94)	5.48 (2.50, 12.02)	0.18 (0.10, 0.32)	35.62 (10.06, 126.08)
< 15	3	0.76 (0.69, 0.84)	0.64 (0.45, 0.82)	2.33 (1.41, 3.85)	0.35 (0.26, 0.46)	8.02 (5.32, 12.10)
≥ 15	3	0.89 (0.85, 0.93)	0.94 (0.89, 0.99)	13.61 (5.92, 31.31)	0.12 (0.07, 0.20)	126.72 (33.10, 485.15)
**Definition of poor response (< 4 oocytes)**						
AMH	11	0.78 (0.70, 0.85)	0.77 (0.69, 0.83)	3.24 (2.50, 4.21)	0.33 (0.24, 0.45)	11.27 (6.62, 19.19)
AFC	9	0.81 (0.74, 0.87)	0.80 (0.73, 0.87)	4.00 (2.76, 5.79)	0.27 (0.19, 0.38)	16.76 (8.76, 30.18)
**Sample size**						
AMH-Poor response						
< 200	23	0.81 (0.75, 0.87)	0.80 (0.73, 0.86)	4.12 (3.00, 5.65)	0.23 (0.17, 0.32)	17.80 (10.54, 20.05)
≥ 200	6	0.74 (0.61, 0.84)	0.83 (0.75, 0.88)	4.26 (3.15, 5.78)	0.32 (0.22, 0.47)	13.45 (8.72, 20.74)
AMH-High response						
< 200	8	0.83 (0.72, 0.91)	0.78 (0.65, 0.87)	3.79 (2.29, 6.26)	0.21 (0.12, 0.37)	17.62 (7.50, 41.44)
≥ 200	11	0.81 (0.73, 0.87)	0.87 (0.79, 0.92)	6.12 (3.63, 10.33)	0.22 (0.15, 0.33)	27.66 (12.24, 62.49)
AFC-Poor response						
< 200	10	0.77 (0.60, 0.88)	0.85 (0.76, 0.91)	5.27 (3.16, 8.79)	0.27 (0.15, 0.50)	19.23 (7.82, 47.30)
≥ 200	5	0.69 (0.50, 0.84)	0.84 (0.73, 0.91)	4.33 (2.86, 6.53)	0.36 (0.22, 0.59)	11.87 (6.83, 20.63)
AFC-high response						
< 200	2	0.89 (0.78, 0.99)	0.70 (0.35, 0.99)	-	-	-
≥ 200	4	0.84 (0.76, 0.92)	0.88 (0.75, 0.99)	6.70 (2.57, 17.45)	0.17 (0.08, 0.37)	39.11 (7.15, 213.98)

*PLR* Positive likelihood ratio, *NLR* Negative likelihood ratio, *DOR* Diagnostic odds ratio

### Accuracy of AMH and AFC for predicting high response

Table 3 presented the pooled predictive ability of AMH and AFC for high response in IVF/ICSI treatments. The meta-analysis’s overall pooled sensitivity and specificity of AMH were 0.81 (95%CI: 0.76–0.86) and 0.84 (95%CI: 0.77–0.90), respectively. Heterogeneity was found in both sensitivity and specificity (*I*^2^ = 83.00% and95.90%, respectively). The overall ROC curve was presented in Fig. 2C, and AUC was 0.89 (95%CI: 0.86–0.91). The overall pooled sensitivity and specificity of AFC were 0.85 (95%CI: 0.77–0.91) and 0.83 (95%CI: 0.64–0.94), respectively. The test for heterogeneity demonstrated that there was significant heterogeneity in both sensitivity and specificity (*I*^2^ = 74.53% and 96.70%, respectively). The overall ROC curve was presented in Fig. 2D, and AUC was 0.90 (95%CI: 0.87–0.92).

### Subgroup analysis

Comparison of the summary estimates for the prediction of poor or high response showed significant difference in performance for AMH compared with AFC [poor (sensitivity: 0.80 vs 0.74, *P* < 0.050; specificity: 0.81 vs 0.85, *P* < 0.001); high (sensitivity: 0.81 vs 0.87, *P* < 0.001)]. There were no significant differences between the AUC of AMH and AFC for predicting high (*P* = 0.835) or poor response (*P* = 0.567). Besides, in the same definition of poor response (< 4 oocytes), AMH and AFC tests had significant differences in sensitivity (0.78 vs 0.81, *P* < 0.001) and specificities (0.77 vs 0.80, *P* < 0.001) (Table 3). However, no significant differences were found between the AUC of AMH and AFC (*P* = 0.800).

### Meta-regression analysis

For AMH, the cut-off value was a significant source of heterogeneity (poor: *P* = 0.020). For AFC, the cut-off value was a significant source of heterogeneity (poor: *P* < 0.010; high: *P* < 0.050). However, sample size was not the significant source of heterogeneity (*P* > 0.05).

### Publication bias

**Fig. 3:**
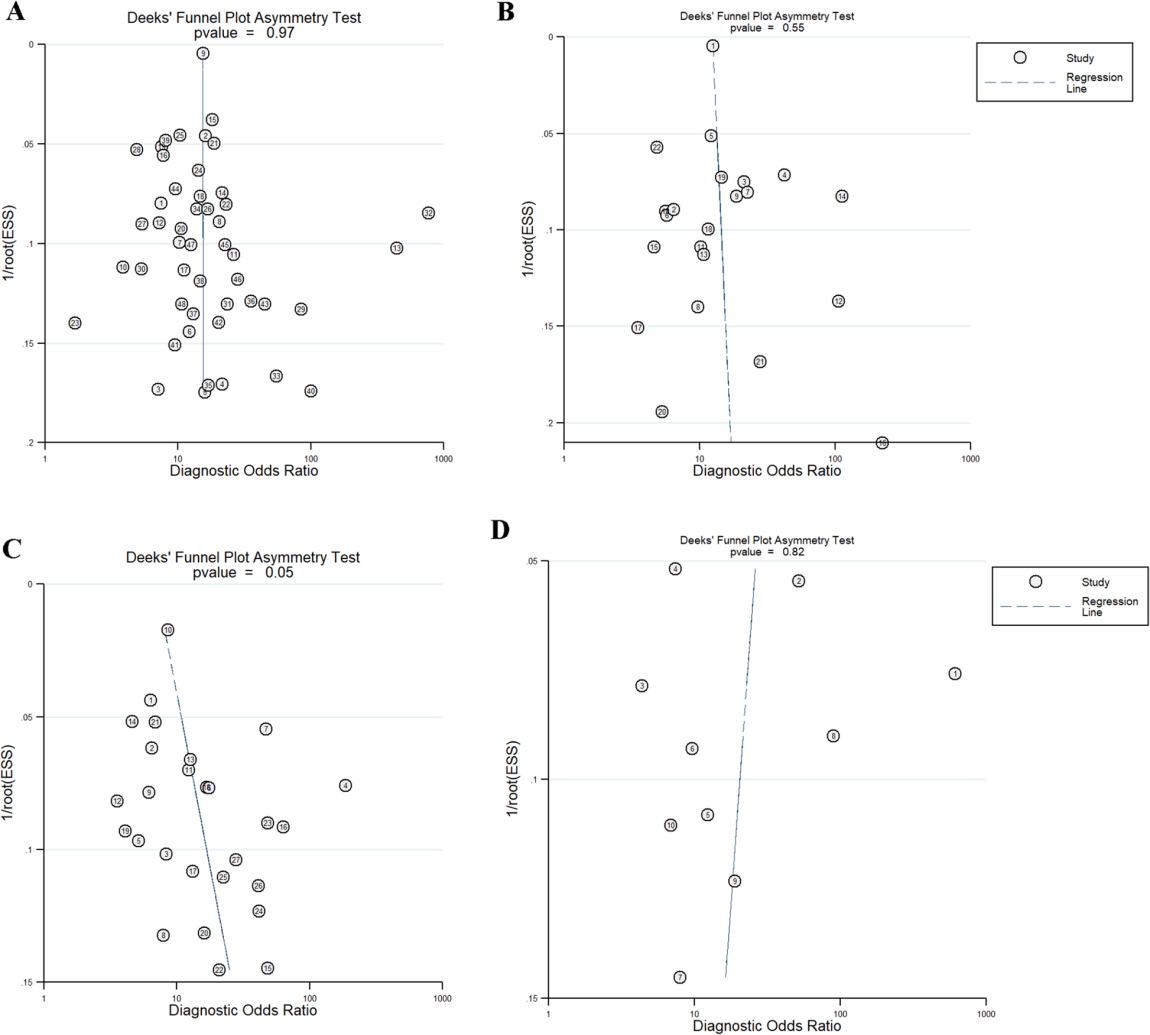
Deek’s funnel plot for the publication bias. **A** AMH-poor response; **B** AFC- poor response; **C** AMH-high response; **D** AFC-high response

Deek’s plot indicated that there was no publication bias in AMH for predicting poor response (*P* = 0.510, Fig. 3A) and high response AFC (*P* = 0.348, Fig. 3C), and AFC for predicting poor (*P* = 0.396, Fig. 3B) and high response (*P* = 0.818, Fig. 3D).

## Discussion

### Main findings

The present meta-analysis summarizes the available evidence about the accuracy of AMH and the AFC for predicting poor or high response to ovarian stimulation in IVF treatments. Although the differences were significant, both AMH and AFC had similar sensitivities and specificities. It seems that both AMH and AFC have a good discriminatory capacity to predict poor or high response in IVF. Besides, the ROC curves did not indicate a better predictive ability for AMH than for AFC, and the difference was not statistically significant. Our results were consistent with previous studies [13, 14, 48, 58]. For example, Broer et al. [13, 14] in their meta-analysis thought that both AMH and AFC are accurate predictors of poor or high response to ovarian hyperstimulation, and both tests appear to have clinical value.

Prior research indicated AFC is better than AMH to predict poor ovarian response [10]. However, several studies argued that the level of AMH is a better predictor of ovarian response than the AFC [11, 43]. In our study, results presented that a comparison of the summary estimates for the prediction of poor or high response showed a significant difference in performance for AMH compared with AFC while there was no significant difference in ROC curves. The discrepancies between studies could be associated with the heterogeneity of the definitions of ovarian response to ovarian stimulation. Therefore, our study conducted a subgroup analysis based on the definition of poor response, and we found that AFC was relatively better than AMH tests in both sensitivity (0.81 vs 0.78, *P* < 0.001) and specificities (0.80 vs 0.77, *P* < 0.001) when the poor response was defined as < 4 oocytes. However, although no significant differences were found in ROC curves, AFC seemed to perform slightly better than AMH for predicting poor response (0.87 vs 0.84). Also, Broer et al. [13] had similar findings in AFC and AMH for the prediction of high response.

Our study found that the accuracy of AMH and AFC for the prediction of poor or high response had many different kinds of cut-off values, which is difficult for clinical practice. Therefore, the present study performed a subgroup analysis based on the range of cut-off values. The accuracy threshold value of AFC for predicting high response achieved the highest AUC when the cut-off value was ≥ 15. The corresponding AUC was 0.90 (95%CI: 0.88, 0.93) with a sensitivity of 0.89 and a specificity of 0.94, which indicates the predictive ability with this interval is higher than the range of cut-off value < 15.

The characteristics of patients could predict abnormal ovarian response, including age, menstrual cycle length, and body mass index. However, these factors have limited predictive value. Therefore, emerging studies reported that the multivariate models predicted ovarian response, and found the model could improve the predictive power [17, 59-61]. For example, Honnma et al. [60] thought that serum AMH in combination with age is a better indicator than AMH alone. Therefore, clinicians should consider patients’ characteristics and biomarkers together to accurately predict ovarian response in IVF treatments.

### Clinical implications

The abnormal response may increase patient discomfort and even decrease the chance of pregnancy. According to the register of the Italian national assisted reproduction technique (ART) in 2010, it reported that 6.7% were canceled due to poor ovarian response, and 1.5% due to ovarian hyperstimulation syndrome (OHSS) in 52,676 IVF cycles [1]. In other words, more than 4300 cycles were canceled every year for an abnormal response to stimulation with gonadotrophins. Furthermore, approximately 35% of couples abandon IVF treatments for physical and psychological burden, and 10% for inadequate ovarian response in the first cycle [62]. Therefore, it is important to reduce the dropout rate in IVF treatments by reducing abnormal responses. Our study found that both AMH and AFC were a good discriminatory capacity to predict poor or high response in IVF. Besides, increasingly studies reported that AMH level is becoming a preferred method for the prediction of ovarian reserve in most women [7, 63]. A multivariable approach, combining patient characteristics and AMH also should be taken into account in the evaluation of ovarian response.

### Limitations

Several limitations would be noted in this meta-analysis. First, relatively high heterogeneity still existed. Although we found that the cut-off value was a significant source of heterogeneity in the present study, heterogeneity was caused by other factors, such as study quality characteristics, and study populations among all included studies. In addition, we found that the quality of the included studies was poor, so more high-quality studies are needed to confirm our conclusions in the future. Second, language bias may exist due to the inclusion of only English articles in the meta-analysis. Third, the predictive value of AMH and AFC for ovarian response was not always assessed in a head-to-head comparison in the same study. The accuracy of the results will be affected to some extent due to the differences in cut-off value and sample size. For this issue, we have tried to enhance the persuasiveness of the paper through meta regression and subgroup analysis.

## Conclusions

In sum, the present meta-analysis demonstrated that both AMH and AFC have a good predictive ability to predict poor or high responses in IVF treatment.

## Supplementary Information

**Additional file 1.**

## Data Availability

All data generated or analyzed during this study are included in this published article.

## Abbreviations

AMH: Anti-Müllerian hormone
ROC: Receiver operator characteristic
COS: Controlled ovarian stimulation
IVF: In vitro fertilization
LH: Luteinizing hormone
FSH: Basal follicle-stimulation hormone
AFC: Antral follicle count
TP: True positives
FP: False positives
FN: False negatives
TN: True negatives
DOR: Diagnostic odds ratio
CIs: Confidence intervals
OHSS: Ovarian hyperstimulation syndrome
