# Supplementary material for: Comparison of anti-Müllerian hormone and antral follicle count in the prediction of ovarian response: a systematic review and meta-analysis
Source: J Ovarian Res. 2023 Jun 27;16:117. doi: 10.1186/s13048-023-01202-5 (PMC10294345; doi:10.1186/s13048-023-01202-5)
Supplement: Supplementary file 1 — Additional file 1. [file 13048_2023_1202_MOESM1_ESM.docx]

Study quality-QUADAS-2

| Study | Risk of Bias | | | | Applicability of Concern | | |
| --- | --- | --- | --- | --- | --- | --- | --- |
|  | Patients  Selection | Index  Test | References  Standard | Flow and  Timing | Patients  Selection | Index  Test | References  Standard |
| Li et al 2016 | UR | LR | UR | UR | LR | LR | UR |
| Fouda et al 2010 | UR | LR | UR | UR | LR | LR | UR |
| Neeta et al 2013 | HR | LR | UR | UR | LR | LR | UR |
| Akbari Sene et al 2021 | HR | LR | UR | HR | LR | LR | UR |
| Izhar et al 2021 | UR | HR | HR | UR | LR | LR | HR |
| Tan et al 2021 | LR | LR | UR | UR | LR | LR | UR |
| Martínez et al 2013 | UR | LR | UR | HR | LR | LR | UR |
| Baker et al 2018 | HR | LR | UR | UR | LR | LR | UR |
| Kamel et al 2014 | UR | HR | HR | HR | LR | LR | HR |
| Fabregues et al 2018 | UR | LR | UR | UR | LR | LR | UR |
| Heidar et al 2015 | UR | LR | UR | UR | LR | LR | UR |
| Ashrafi et al 2016 | UR | LR | UR | UR | LR | LR | UR |
| Vembu et al 2017 | UR | LR | UR | UR | LR | LR | UR |
| Neves et al 2020 | UR | LR | UR | UR | LR | LR | UR |
| Islam et al 2015 | UR | LR | UR | UR | LR | LR | UR |
| Baker et al 2021 | UR | LR | UR | UR | LR | LR | UR |
| Palhares et al 2014 | LR | LR | UR | HR | LR | LR | UR |
| Frattarelli et al 2003 | HR | LR | UR | UR | LR | LR | UR |
| Jayaprakasan et al 2010 | LR | LR | UR | HR | LR | LR | UR |
| Tolikas et al 2011 | UR | LR | UR | UR | LR | LR | UR |
| Tremellen et al 2005 | HR | LR | UR | UR | LR | LR | UR |
| Kunt et al 2011 | UR | LR | UR | HR | LR | LR | UR |
| Marca et al 2007 | UR | LR | UR | HR | LR | LR | UR |
| Mutlu et al 2013 | UR | LR | UR | UR | LR | LR | UR |
| Peñarrubia et al 2005 | UR | LR | UR | UR | LR | LR | UR |
| Nardo et al 2009 | HR | LR | UR | UR | LR | LR | UR |
| Jayaprakasan et al 2007 | LR | LR | UR | HR | LR | LR | UR |
| Eldar-Geva et al 2005 | HR | LR | UR | UR | LR | LR | UR |
| Fiçicioglu et al 2006 | UR | LR | UR | UR | LR | LR | UR |
| McIlveen et al 2007 | UR | LR | UR | HR | LR | LR | UR |
| Bancsi et al 2004 | HR | LR | UR | HR | LR | LR | UR |
| Muttukrishna et al 2004 | UR | LR | UR | UR | LR | LR | UR |
| Nakhuda et al 2007 | UR | HR | HR | HR | LR | LR | HR |
| Aflatoonian et al 2009 | HR | LR | UR | UR | LR | LR | UR |
| Yong et al 2003 | HR | LR | UR | UR | LR | LR | UR |
| Järvelä et al 2003 | LR | LR | UR | UR | LR | LR | UR |
| Gnoth et al 2008 | UR | LR | UR | UR | LR | LR | UR |
| Lee et al 2008 | UR | HR | HR | HR | LR | LR | HR |
| Nelson et al 2007 | HR | LR | UR | HR | LR | LR | UR |
| van Rooij et al 2002 | HR | LR | UR | HR | LR | LR | UR |
| Soldevila et al 2007 | LR | LR | UR | HR | LR | LR | UR |
| Lee et al 2011 | UR | HR | HR | HR | LR | LR | HR |

LR, low risk; HR, high risk; UR, unclear risk.

**Search strategy**

**PubMed**

| **PubMed** |  | **Search strategy** | **Numbers** |
| --- | --- | --- | --- |
| **Patient** | **#1** | "Fertilization in Vitro"[Mesh] | 39254 |
|  | **#2** | ((((((((((((((((In Vitro Fertilization[Title/Abstract]) OR (In Vitro Fertilizations[Title/Abstract])) OR (Test-Tube Fertilization[Title/Abstract])) OR (Fertilization, Test-Tube[Title/Abstract])) OR (Fertilizations, Test-Tube[Title/Abstract])) OR (Test Tube Fertilization[Title/Abstract])) OR (Test-Tube Fertilizations[Title/Abstract])) OR (Fertilizations in Vitro[Title/Abstract])) OR (Test-Tube Babies[Title/Abstract])) OR (Babies, Test-Tube[Title/Abstract])) OR (Baby, Test-Tube[Title/Abstract])) OR (Test Tube Babies[Title/Abstract])) OR (Test-Tube Baby[Title/Abstract])) OR (in vitro fertilisation[Title/Abstract])) OR (assisted[Title/Abstract])) OR (intracytoplasmic[Title/Abstract])) OR (intracytoplasmatic[Title/Abstract]) | 270514 |
|  | **#3** | **#1 OR #2** | 285879 |
| **Intervention** | **#4** | "Anti-Mullerian Hormone"[Mesh] | 3731 |
|  | **#5** | (((((((((((((((((Anti Mullerian Hormone[Title/Abstract]) OR (Mullerian-Inhibiting Factor[Title/Abstract])) OR (Mullerian Inhibiting Factor[Title/Abstract])) OR (Anti-Mullerian Factor[Title/Abstract])) OR (Anti Mullerian Factor[Title/Abstract])) OR (Mullerian-Inhibitory Substance[Title/Abstract])) OR (Mullerian Inhibitory Substance[Title/Abstract])) OR (Mullerian Inhibiting Hormone[Title/Abstract])) OR (Mullerian Inhibiting Substance[Title/Abstract])) OR (Mullerian Regression Factor[Title/Abstract])) OR (Mullerian-Inhibiting Hormone[Title/Abstract])) OR (Anti-Muellerian Hormone[Title/Abstract])) OR (Anti Muellerian Hormone[Title/Abstract])) OR (Hormone, Anti-Muellerian[Title/Abstract])) OR (Antimullerian Hormone[Title/Abstract])) OR (AMH[Title/Abstract])) OR (antral follicle count[Title/Abstract])) OR (AFC[Title/Abstract]) | 9070 |
|  | **#6** | **#4 OR #5** | 9288 |
| **P+I** | **#7** | **#3 AND #6** | 1676 |

**Cochrane Library**

| Cochrane |  | Search strategy | Numbers |
| --- | --- | --- | --- |
| Patient | **#1** | MeSH descriptor: [Fertilization in Vitro] explode all trees | 2276 |
|  | **#2** | (In Vitro Fertilization):ti,ab,kw OR(In Vitro Fertilizations):ti,ab,kw OR(Test-Tube Fertilization):ti,ab,kw OR(Fertilization, Test-Tube):ti,ab,kw OR(Fertilizations, Test-Tube):ti,ab,kw OR(Test Tube Fertilization):ti,ab,kw OR(Test-Tube Fertilizations):ti,ab,kw OR(Fertilizations in Vitro):ti,ab,kw OR(Test-Tube Babies):ti,ab,kw OR(Babies, Test-Tube):ti,ab,kw OR(Baby, Test-Tube):ti,ab,kw OR(Test Tube Babies):ti,ab,kw OR(Test-Tube Baby):ti,ab,kw OR(in vitro fertilisation):ti,ab,kw OR(assisted):ti,ab,kw OR(intracytoplasmic):ti,ab,kw OR(intracytoplasmatic):ti,ab,kw | 41588 |
|  | **#3** | **#1** OR **#2** | 41588 |
| Intervention | **#4** | (anti⁃Mullerian hormone ):ti,ab,kw OR(Anti Mullerian Hormone):ti,ab,kw OR(Mullerian-Inhibiting Factor):ti,ab,kw OR(Mullerian Inhibiting Factor):ti,ab,kw OR(Anti-Mullerian Factor):ti,ab,kw OR(Anti Mullerian Factor):ti,ab,kw OR(Mullerian-Inhibitory Substance):ti,ab,kw OR(Mullerian Inhibitory Substance):ti,ab,kw OR(Mullerian Inhibiting Hormone):ti,ab,kw OR(Mullerian Inhibiting Substance):ti,ab,kw OR(Mullerian Regression Factor):ti,ab,kw OR(Mullerian-Inhibiting Hormone):ti,ab,kw OR(Anti-Muellerian Hormone):ti,ab,kw OR(Anti Muellerian Hormone):ti,ab,kw OR(Hormone, Anti-Muellerian):ti,ab,kw OR(Antimullerian Hormone):ti,ab,kw OR(AMH):ti,ab,kw OR(antral follicle count):ti,ab,kw OR (AFC):ti,ab,kw | 1475 |
| P+I+O | **#5** | **#3** AND **#4** | 520 |

**Embase**

| Embase |  | Search strategy | Numbers |
| --- | --- | --- | --- |
| Patient | **#1** | 'in vitro fertilization'/exp | 104708 |
|  | **#2** | 'Fertilization in Vitro':ab,ti or 'In Vitro Fertilization':ab,ti or 'In Vitro Fertilizations':ab,ti or 'Test-Tube Fertilization':ab,ti or 'Fertilization, Test-Tube':ab,ti or 'Fertilizations, Test-Tube':ab,ti or 'Test Tube Fertilization':ab,ti or 'Test-Tube Fertilizations':ab,ti or 'Fertilizations in Vitro':ab,ti or 'Test-Tube Babies':ab,ti or 'Babies, Test-Tube':ab,ti or 'Baby, Test-Tube':ab,ti or 'Test Tube Babies':ab,ti or 'Test-Tube Baby':ab,ti or 'in vitro fertilisation':ab,ti or 'assisted':ab,ti or 'intracytoplasmic':ab,ti or 'intracytoplasmatic':ab,ti | 333998 |
|  | **#3** | **#1 OR #2** | 391278 |
| Intervention | **#4** | 'muellerian inhibiting factor'/exp | 8661 |
|  | **#5** | 'anti⁃mullerian hormone':ab,ti OR 'anti mullerian hormone':ab,ti OR 'mullerian-inhibiting factor':ab,ti OR 'mullerian inhibiting factor':ab,ti OR 'anti-mullerian factor':ab,ti OR 'anti mullerian factor':ab,ti OR 'mullerian-inhibitory substance':ab,ti OR 'mullerian inhibitory substance':ab,ti OR 'mullerian inhibiting hormone':ab,ti OR 'mullerian inhibiting substance':ab,ti OR 'mullerian regression factor':ab,ti OR 'mullerian-inhibiting hormone':ab,ti OR 'anti-muellerian hormone':ab,ti OR 'anti muellerian hormone':ab,ti OR 'hormone, anti-muellerian':ab,ti OR 'antimullerian hormone':ab,ti OR 'amh':ab,ti OR 'antral follicle count':ab,ti OR 'afc':ab,ti | 15143 |
|  | **#6** | **#4 OR #5** | 17087 |
| P+I | **#7** | **#3 AND #6** | 5131 |
